# Bergamo Field Hospital Confronting COVID-19: Operating Instructions

**DOI:** 10.1017/dmp.2020.447

**Published:** 2020-11-19

**Authors:** Ornella Spagnolello, Silvia Rota, Oliviero Francesco Valoti, Claudio Cozzini, Pietro Parrino, Gina Portella, Martin Langer

**Affiliations:** 1 Bergamo field hospital, ASST Papa Giovanni XXIII Hospital of Bergamo, Bergamo, Italy; 2 Department of Public Health and Infectious Diseases, University of Rome Sapienza, Rome, Italy; 3 Department of Basic and Clinical Neuroscience, Institute of Psychiatry, Psychology and Neuroscience, King’s College London, London, UK; 4 EMERGENCY-NGO, Milan, Italy

**Keywords:** COVID-19, field hospital, infection prevention and control measures, pandemic

## Abstract

The coronavirus disease (COVID-19) pandemic represented an unprecedented challenge for health care facilities, and innovative solutions were urgently required to overcome the high volume of critically ill infectious patients, limit in-hospital outbreaks, and limit the risk of occupational infection for health care workers (HCWs). Bergamo was the hardest-hit Italian province by COVID-19, and the local health care system had to undergo a profound and prompt reorganization. A COVID-19-only field hospital was rapidly set up meeting the standards for severe acute respiratory infection (SARI) treatment centers (https://apps.who.int/iris/handle/10665/331603). A zones partition, dedicated in-hospital pathways for HCWs, strict infection prevention and control (IPC) measures, and constant staff supervision were key components of our strategy to limit the risk of occupational infection for HCWs. Herein, we present the Bergamo field hospital layout enlightening fundamental IPC measures adopted as a valuable example of a SARI treatment center confronting COVID-19.

## Introduction

Coronavirus disease (COVID-19) was confirmed a pandemic by the World Health Organization (WHO) on March 11, 2020. Lombardy was the hardest-hit Italian region, and the highest number of patients was recorded in Bergamo Province (about 1 115 000 inhabitants) with 12 294 cases as of May 12, 2020.^1^

It is postulated that the main way of transmission of severe acute respiratory syndrome coronavirus 2 (SARS-CoV-2) is through droplets exhaled by a carrier, which can travel up to 1 meter and contaminate surfaces, representing a serious infectious hazard for health care workers (HCWs). Personal protective equipment (PPE) and patient’s isolation are crucial in the management of COVID-19 patients, in order to protect staff and, indirectly, the community. Unfortunately, by the time that Italy was facing the COVID-19 epidemic peak, almost 10% of total cases reported were among HCWs.^2^

With the COVID-19 outbreak spreading out in Bergamo, a high volume of critically sick patients rapidly outpaced the local hospitals’ capacity.^3^ Only patients with severe respiratory symptoms were admitted to hospitals after a highly selective triage. Local health care facilities underwent a profound reorganization and a COVID-19-only field hospital was rapidly set up.

Herein, we illustrate the Bergamo field hospital (BFH) layout designed under the supervision of the EMERGENCY team, an Italian non-governmental organization that confronted the Ebola outbreak in Sierra Leone in 2014–2015.^4^

## Bergamo Field Hospital

**FIGURE 1.**
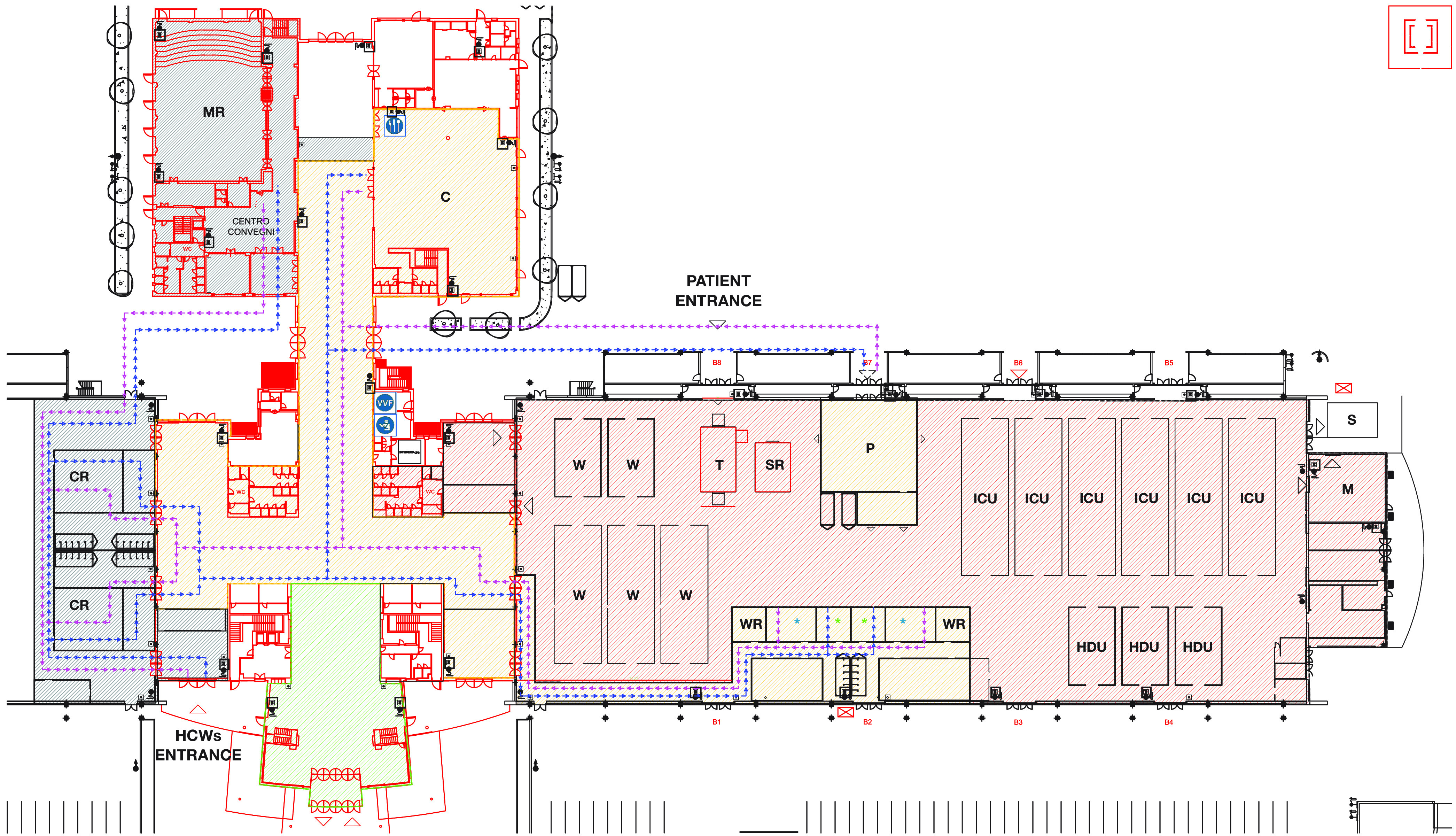
Bergamo field hospital layout. This figure illustrates the BFH layout and HCWs’ pathways. The gray, green, and yellow zones are reported accordingly. Blue arrows point to HCWs’ incoming path, whereas violet arrows indicate the exit path. T = triage; SR = shock room; P = pharmacy; W = ward; ICU = intensive care unit; HDU = high-dependency unit; M = morgue; CR = changing room; 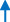 MR = meeting room; 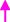 C = canteen; WR = working room; 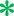 (green asterisk) = donning room; 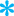 (blue asterisk) = doffing room; HCWs’ incoming pathway; HCWs’ exit path.

The BFH is a 142-bed capacity hospital set up in a trade fair center, a 21.000-m^2^ building strategically located in the outskirts of Bergamo. The hospital layout can be found in Figure 1. The BFH offered 72 additional intensive care beds, 30 high-dependency beds, and 40 ward beds to the ASST Papa Giovanni XXIII Hospital of Bergamo.

The conversion of the pre-existing building into the fully equipped BFH started while Bergamo was facing the COVID-19 epidemic peak and was accomplished in 10 consecutive days.^5^ Meeting the standards set by the WHO for a severe acute respiratory infection (SARI) treatment center,^6^ the BFH became a COVID-19 hub gathering patients with a confirmed diagnosis of COVID-19.

The technical division of the EMERGENCY team was the core in planning the BFH layout.

## Hospital Layout

As shown in Figure 1, the BFH is mainly divided into a staff and a patients’ area. The former is further divided into 3 zones: green, gray, and yellow. Whereas the green zone is exclusively allocated to administrative/logistic staff, only staff dedicated to clinical activities have access to the gray and yellow zones.

The patients’ area is uniformly flagged as the red zone, where any level of care is delivered. Here, patients are assigned to 2 main areas in relation to the chance of undergoing aerosol-generating procedures: the ward area on the left wing and the intensive and high-dependency units on the right wing. Despite being divided in 14 sectors, the red zone is conceived as an open space in order to facilitate teams’ cooperation and therefore minimize the time required for patients to step from 1 level of care to another. COVID-19 patients have access to the red zone from the triage, where the disease severity is clinically assessed so that patients are assigned to a specific sector accordingly. For highly critical cases, a shock room is located nearby.

A physical border separates the yellow zone from the red one. Upon this border are located respectively, 2 PPE donning and doffing rooms along with 2 working areas, 1 of each serving 1 specific wing. This minimizes contact between HCWs in charge of each wing. The PPE donning and doffing rooms are the only **gateways to** the red zone. On the contrary, the working areas are dedicated to staff employed in activities not in direct contact with patients. At this level, the border is mostly made by transparent surfaces in order to enable visual contact and monitor activities running in the red zone. Here, workers wear a surgical mask and not other PPE.

The pharmacy is located in an isolated sector in the red zone with an outside connection for items delivery and staff entrance. A drugs collection occurs through a gravity chute – a delivering system that avoids air recirculation, maintaining security distances. In this manner, pharmacy staff can wear surgical masks only.

A top-down mechanical ventilation system consisting in pre-installed ceiling fans enables 6 times air changes per hour across the red zone, creating a continuous airflow from the edge of the yellow zone toward extraction fans located on the north face of the BFH (airflow direction from clean to dirty zones). Exhaled air is then treated through UV lamps before being released outside.

## Health Care Workers Flow

### Gray Zone

The staff entrance and changing room together constitute the gray zone. The staff entrance is under constant surveillance with a guard ensuring temperature screening and checking for mask wearing and handwashing of all authorized people entering. In case of fever detection, a 2-entrance room with a buffer area for clinical assessment is located nearby.

Male and female changing rooms are spacious enough to avoid overcrowding during shift changes. Here, staff members deposit their clothes and personal belongings, perform hand hygiene, and step into the next room to wear cleaned scrubs and sanitized clogs before entering the yellow zone.

A big meeting room is also part of the gray zone.

### Yellow Zone

The yellow zone embraces facilities like restrooms and the hospital canteen, designed wide enough to observe social distancing. A mandatory pathway leads then to the donning rooms. Each donning room is a 4-people capacity room fully equipped with PPE items, 2 mirrors, and a door opening outward on the red zone. At this step, a team of trained infection prevention and control (IPC) officers constantly supervise staff members wearing PPE properly before entering the red zone.

### Red Zone

Once in the red zone, HCWs are asked to observe a list of IPC recommendations. No restrooms for staff are available to minimize physical exposure to contaminated surfaces.

Patients, where feasible, wear a surgical mask to minimize environmental contamination.

### Yellow Zone

For HCWs, the only way out from the red zone is though the doffing rooms. Here, the process of removing contaminated PPE takes place under the surveillance of IPC officers. Mirrors, PPE waste disposals, sinks, soaps, and hand sanitizers are meticulously placed to allow HCWs to safely remove PPE and get decontaminated. Despite the urgent need of removing PPE worn for long hours, this is the staff’s most dangerous step of the day, and IPC officers’ supervision is paramount to remember the importance of being accurate. Contaminated objects, such as glasses, are carefully decontaminated and surgical masks are provided.

On the way to the changing rooms, HCWs remove uniforms and clogs and take a shower.

## Discussion

Facing the COVID-19 pandemic, HCWs and policy-makers have been forced to change their way of delivering health assistance, looking at past experiences of less developed countries as a model at times.^7^

HCWs are at high risk for secondary infection. Indeed, 25 937 frontline HCWs tested positive for SARS-CoV-2 by the middle of May 2020 in Italy.^8^ This had an extraordinary impact on physical and mental stress of medical staff, already overwhelmed by the exceptional situation.^9^

Bergamo was heavily affected by this global emergency, which led to a deep structural and logistical reorganization of the local health care system. In this scenario, the BFH was urgently set up in compliance with recently issued WHO’s list of recommendations and technical guidelines about how to structure and operate SARI treatment centers.

SARI treatment centers are part of the strategic priorities to counteract acute respiratory infections that have the potential for rapid spread resulting in an epidemic or pandemic. In fact, during an epidemic or pandemic, the institution of SARI treatment centers is the only way to prevent health care systems from being overwhelmed, to simplify the referral pathway, and to minimize exposure for HCWs, patients, and the community.

The EMERGENCY team was crucial in implementing the WHO standards in the BFH layout and promoting new professionals as key IPC components, such as the IPC officers’ team. Indeed, despite the numerous debates ongoing about PPE-type and availability, the effectiveness of PPE strongly depends on adequate staff training, appropriate hand hygiene, and appropriate human behavior. Workers unaccustomed to PPE are more likely to use it incorrectly and thus put themselves at higher risk of infection, particularly when putting on and taking off their PPE. In our experience, layperson volunteers also successfully fulfilled the role of IPC officers after being trained in IPC measures and sanitation procedures. This enabled the service to run 24/7, despite the resource-limited scenario.

## Conclusion

In conclusion, field hospitals play a key role during an epidemic and pandemic. Such was the case for many field hospitals established as Ebola treatment centers during the epidemic of Ebola in Western Africa.^10^ A zones partition and mandatory pathways for HCWs are crucial to optimize PPE availability and minimize staff exposure. Moreover, in our frontline experience, the contribution of the IPC officers’ team was relevant in supervising staff. We firmly believe that this might have yielded a valuable contribution in further reducing the risk of contamination and boosting safety self-awareness in HCWs.

The measures taken by the BFH during the COVID-19 pandemic may not be universally applicable elsewhere. However, the presented IPC measures have been practically executed and can be a source of inspiration in future emergencies.
